# Electrochemical Aptasensing Platform for the Detection of Retinol Binding Protein-4

**DOI:** 10.3390/bios14020101

**Published:** 2024-02-16

**Authors:** Kamila Malecka-Baturo, Paulina Żółtowska, Agnieszka Jackowska, Katarzyna Kurzątkowska-Adaszyńska, Iwona Grabowska

**Affiliations:** 1 Institute of Animal Reproduction and Food Research Polish Academy of Sciences, Tuwima 10, 10-748 Olsztyn, Poland; k.malecka@pan.olsztyn.pl (K.M.-B.); k.kurzatkowska-adaszynska@pan.olsztyn.pl (K.K.-A.); 2 Department of Chemistry, University of Warmia and Mazury, Plac Łódzki 4, 10-721 Olsztyn, Poland; paulina.zoltowska2002@gmail.com (P.Ż.); agnieszka.jackowska123@gmail.com (A.J.)

**Keywords:** electrochemical aptasensor, optimization process, retinol binding protein-4, RBP-4

## Abstract

Here, we present the results of our the electrochemical aptasensing strategy for retinol binding protein-4 (RBP-4) detection based on a thiolated aptamer against RBP-4 and 6-mercaptohexanol (MCH) directly immobilized on a gold electrode surface. The most important parameters affecting the magnitude of the analytical signal generated were optimized: (i) the presence of magnesium ions in the immobilization and measurement buffer, (ii) the concentration of aptamer in the immobilization solution and (iii) its folding procedure. In this work, a systematic assessment of the electrochemical parameters related to the optimization of the sensing layer of the aptasensor was carried out (electron transfer coefficients *(α),* electron transfer rate constants (*k*^0^) and surface coverage of the thiolated aptamer probe (*Γ_Apt_*)). Then, under the optimized conditions, the analytical response towards RBP-4 protein, in the presence of an Fe(CN)_6_^3−/4−^ redox couple in the supporting solution was assessed. The proposed electrochemical strategy allowed for RBP-4 detection in the concentration range between 100 and 1000 ng/mL with a limit of detection equal to 44 ng/mL based on electrochemical impedance spectroscopy (EIS). The specificity studies against other diabetes biomarkers, including vaspin and adiponectin, proved the selectivity of the proposed platform. These preliminary results will be used in the next step to miniaturize and test the sensor in real samples.

## 1. Introduction

Electrochemical biosensors have been of great research interest for many years, mainly because they are characterized by high sensitivity, ease of use with simple equipment, relatively low cost and rapid readouts. Thus, they can be integrated with portable, wearable and implantable systems for point-of-care diagnostic purposes [1]. Moreover, among the different recognition elements used in the construction of electrochemical biosensors, aptamers have recently gained a lot of scientific attention. Aptamers, ssDNA or ssRNA strands can be produced by the combinatorial chemistry technique SELEX (systematic evolution of ligands by exponential enrichment). They undergo conformational changes after target recognition and binding, accompanied by hydrogen bonding, electrostatic, hydrophobic and Van der Waals interactions, base stacking and intercalation [2]. So far, many examples of the use of electrochemical aptasensors, not only in clinical diagnostics but also in other fields, have been presented in the scientific literature [3,4,5,6,7,8,9]. 

Retinol binding protein 4 (RBP-4), alongside glycated albumin (GA), glycated human serum albumin (GHSA) and the GHSA to human serum albumin (HSA) ratio, belongs to a group of specific biomarkers that can predict the diabetes condition even better than glucose [4]. Moreover, this protein is also considered as a very important biomarker for the prognosis of this disease at early stages [10]. Since Torabi and co-workers have demonstrated that RBP-4 can be detected by a 76-mer ssDNA aptamer [11], a few aptasensors for monitoring RBP-4 have been presented so far [4]. However, their signal transduction mechanisms are based on chemiluminescence [12], Surface Plasmon Resonance (SPR) [13], Enzyme-Linked Antibody–Aptamer Sandwich (ELAAS) [14] and colorimetric gold nanoparticle-based aptasensing [15]. Only one available example of electrochemical biosensor for detection of RBP-4 based on electrochemical impedance spectroscopy (EIS) was presented, but in this case an antibody against RBP-4 was used [16]. No information has been found about an electrochemical aptasensor for the detection of RBP-4 so far. 

The development of an appropriate aptasensing platform is crucial for the reliable detection of specific analytes with this tool. The proper immobilization conditions for an aptamer on the surface of the sensor affects its molecular recognition ability and therefore its sensitivity and selectivity [17,18]. At least three the most important variables should be tested when new aptasensing platform is designed, including, (i) the presence of magnesium in immobilization and the supporting solution, (ii) the concentration of aptamer in the immobilization solution and (iii) the aptamer folding procedure. 

ssDNA aptamers are negatively charged thanks to the presence of numerous anionic phosphate groups. Depending on the environmental conditions, an aptamer molecule can reversibly take on a folded or unfolded conformation. If negative charges are not screened and phosphates are repulsed, aptamer molecules are linear (unfolded). When the negative charges are neutralized, these particles can form intramolecular H bonds and become folded in conformation in the different types of loops and/or knots [19]. Thus, in the formation of stable complexes between proteins and an aptamer, metal ions play an important role. Mg^2+^_,_ K^+^ and Na^+^ influence the folding of DNA or RNA aptamer molecules into their three-dimensional structure, enabling them to bind to their targets and consequently affecting their stability and binding affinity [20,21]. It was proven that even the smallest changes in the concentration of magnesium chloride in the buffer solution affect the affinity of the aptamer to its target, as shown in the thrombin aptamer example [22]. 

The proper density and orientation of aptamer molecules on the surface of the biosensor electrode should provide their specific interaction with freely diffusing analytes. As a consequence, the proper deposition of aptamer molecules on the surface of the electrode is crucial for aptasensor efficiency [18,23,24]. Apart from buffer components, aptamer concentration in immobilization solutions is the next variable influencing the attachment conditions [25]. The surface density of aptamers on the surface of gold electrode could be easily controlled by the thiol–aptamer and diluent molar ratios in the solution [26] in order to reach the optimum surface density. 

As compared with antibodies, aptamers also require an additional step to fold them into a 3D shape based on the denaturation of single-stranded species by heating step. The standard protocol for DNA aptamers involves heating the sample up to 85°C for a few minutes, followed by incubation at room temperature or on ice [27]. 

Taking the above into account, and the fact that each aptamer–analyte pair has its own specific conditions for interactions, in this work, we have undertaken a new task in developing the basis of an aptasensor platform for the electrochemical detection of RBP-4. The aptasensor platform was fabricated via the formation of a self-assembled monolayer containing an aptamer and MCH on a gold surface [24,28]. Our aim was to clarify the effect of MgCl_2_’s presence, the procedure of aptamers molecules folding into their tertiary structure and the concentration of aptamers in the immobilization buffer on the sensitivity of electrochemical aptasensor towards RBP-4 protein. The interaction between the aptamer specific to RBP-4 deposited on the surface of the electrode and RBP-4 was monitored by cyclic voltammetry (CV), square-wave voltammetry (SWV) and EIS in the presence of an [Fe(CN)_6_]^3−/4−^ redox probe. 

## 2. Materials and Methods

### 2.1. Chemicals

MCH, K_3_[Fe(CN)_6_] and K_4_[Fe(CN)_6_], PBS buffer and MgCl_2_ were purchased from Sigma-Aldrich (Poznan, Poland). Potassium hydroxide, sulfuric acid, ethanol and methanol were obtained from POCh (Gliwice, Poland). Alumina slurries with particle sizes of 0.3 and 0.05 µm were produced by Buehler (Lake Bluff, IL, USA). The Apt-RBP-4 sequence of: 5’-ACA GTA GTG AGG GGT CCG TCG TGG GGT AGT TGG GTC GTG G-3’, functionalized with a thiol group (-SH) at its 5’-end, was purchased from Biomers (Ulm, Germany). The aptamer sequence was taken from a paper by Lee and co-workers. RBP-4 was obtained from Abcam (ab111460). Vaspin (SRP4915) and adiponectin (SRP4901) were purchased from Sigma-Aldrich. All aqueous solutions were prepared with deionized and charcoal-treated water (resistivity of 18.2 MΩ cm) filtered with a Milli-Q reagent-grade water system (Millipore, Bedford, MA, USA). Before each measurement, all solutions were purged with nitrogen (ultrapure 6.0, Air Products, Warsaw, Poland) for 10 min in order to remove oxygen. 

### 2.2. Electrochemical Measurements

The electrochemical measurements, including CV, SWV, EIS, were performed using a potentiostat-galvanostat (Metrohm Autolab, Barendrecht, The Netherlands) controlled by NOVA 2.1 software. A three-electrode cell consisted of a gold disk electrode with a 2 mm diameter as a working electrode, the silver chloride electrode (Ag/AgCl) as a reference electrode and a platinum wire as an auxiliary electrode. Gold electrodes were purchased from Bioanalytical Systems (BASi, West Lafayette, IN, USA). These electrodes were characterized by electroactive surface area (A_eas_) of 0.43 ± 0.03 cm^2^ and roughness factor (*RF*) of 13.7 ± 1.1 (Supplementary Materials) [29,30,31]. 

The measurements were performed in (i) 0.1 M PBS buffer or (ii) 0.1 M PBS buffer containing 2 mM MgCl_2_, pH 7.4, in the presence of 1 mM [Fe(CN)_6_]^3−^/[Fe(CN)_6_]^4−^ redox-pair solution, respectively. The electrode responses were expressed as:

- Δ*E* = (*E_n_* − *E*_0_)/*E*_0_ × 100%;
- Δ*I* = (*I_n_* − *I*_0_)/*I*_0_ × 100%;
- Δ*R* = (*R_n_* − *R*_0_)/*R*_0_ × 100%;

where *E_n_* is the difference in the potentials of the oxidation and reduction peaks, *I_n_* is the peak current and *R_n_* is the charge transfer resistance registered in the presence of particular concentration of RBP-4, respectively; *E*_0_ is the difference in the potentials of the oxidation and reduction peaks, *I*_0_ is the peak current and *R*_0_ is the charge transfer resistance registered before electrode contact with RBP-4, respectively.

### 2.3. Fabrication of Electrochemical Aptasensor 

Before modification, each gold electrode surface (Au) was manually polished on flat pads for 5 min with alumina slurries with particle sizes of 0.3 and 0.05 µm, respectively, and carefully washed with Milli-Q water. Electrochemical cleaning of the Au electrodes was then carried out using the CV method. It consisted of two stages: (i) cleaning in 0.5 M KOH solution by cyclic sweeping of the potential range between −0.4 and −1.2 V (vs. Ag/AgCl reference electrode) with a scanning speed of 0.1 Vs^−1^ (3, 50 and 5 cycles) and (ii) in 0.5 M H_2_SO_4_ by sweeping the potential from −0.3 to +1.5 V at 0.1 Vs^−1^ (3, 10 and 5 scans). Finally, the Au surfaces of the working electrodes were electrochemically treated with 10 cycles of CV in the potential range from −0.4 to –1.2 V in 0.5 M KOH. After electrochemical cleaning was complete, each electrode was thoroughly rinsed with Milli-Q water and stored in water for several minutes until the modification step. The surfaces of the gold electrodes prepared in this way were further modified according to the following protocol based on two steps of modifications:

(1) The solutions of Apt-RBP-4 (0.1, 1.0, or 10.0 µM) were either incubated at 70 °C in a water bath for 1 min (prepared in PBS or PBS + 2 mM MgCl_2_) and then exposed to ice cooling (10 min) (called “aptamer folding”) or were not treated in this way (“aptamer without folding”). Then, a 10 μL droplet of solution containing an appropriate concentration of thiolated aptamer (0.1 or 1.0 or 10.0 µM), together with 0.1 µM of MCH, was co-immobilized by drop-casting on the Au electrode, at room temperature, in suitable buffer, for 3 h. The Au/Apt-RBP-4/MCH electrode was rinsed with buffer (PBS or PBS + 2 mM MgCl_2_).
(2) The second step was based on the incubation of modified electrodes in the first stage by dropwise application of 1 mM of MCH solution (either in PBS or in PBS + 2 mM MgCl_2_), for 30 min, in order to cover the non-active sites of the Au/Apt-RBP-4/MCH surface. Then, after rinsing with the same buffer, the electrodes were left overnight in a buffer solution at 4 °C until use.

Upon the modification of Au/Apt-RBP-4/MCH electrodes, they were incubated in 1 μg/mL of RBP-4 in the same buffer as the modification buffer for 1h at room temperature, followed by CV, SWV and EIS measurements.

## 3. Results and Discussion

### 3.1. Optimization of the Folding Procedure, Modification and Supporting Electrolyte Composition

**Figure 1:**
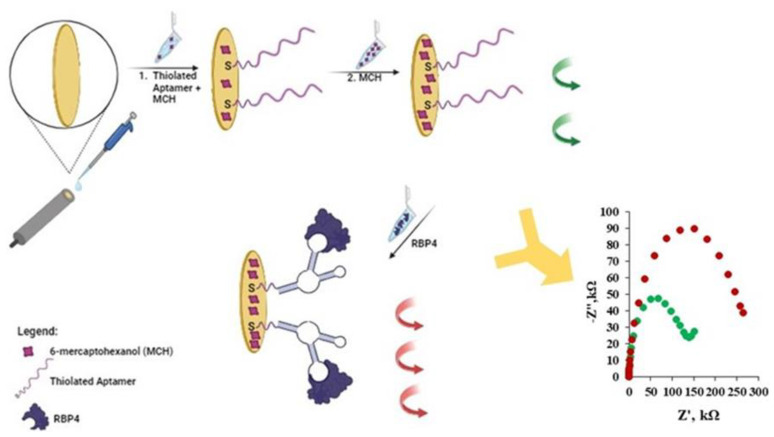
Illustration of gold electrode modification with thiolated aptamer and MCH along with a scheme of analytical signal generation based on aptasensor interaction with RBP-4 protein. Impedance spectra: (●) Apt-RBP-4/MCH modified gold electrode, before interaction with RBP-4 and (●) after interaction with 1 µg/mL of RBP-4.

In this work, we have concentrated on the covalent attachment of an aptamer with a -SH group to the gold surface of an electrode. We applied the two-step immobilization of the thiolated aptamer towards RBP-4, together with MCH acting as filler [32]. The first step consisted of the simultaneous co-immobilization of the thiolated aptamer and MCH, and the second step included backfilling with MCH. Such modified electrodes were treated with a 1 µg/mL solution of RBP-4 protein and then CV, SWV and EIS, expressed as relative changes in signals registered before and upon electrode contact with RBP-4 protein, were measured. A scheme of the gold electrode modification with the thiolated aptamer molecules and MCH, along with a scheme of the analytical signal generation based on the aptasensor’s interaction with proteins, is illustrated in Figure 1. Generally, the detection of RBP-4 protein was confirmed by CV, SWV and EIS experiments using [Fe(CN)_6_]^3−/4−^ as a redox probe present in the supporting electrolyte. The idea behind using this type of biosensor is based on the phenomenon of changing the availability of [Fe(CN)_6_]^3−/4−^ to the solid support which is related to the binding of the analyte to the appropriately modified solid electrode surface [32,33].

Experiments with positively charged redox-active indicator – hexaammineruthenium (II) chloride were also performed (more details in Supplementary Materials) [34,35,36,37]. 

**Figure 2:**
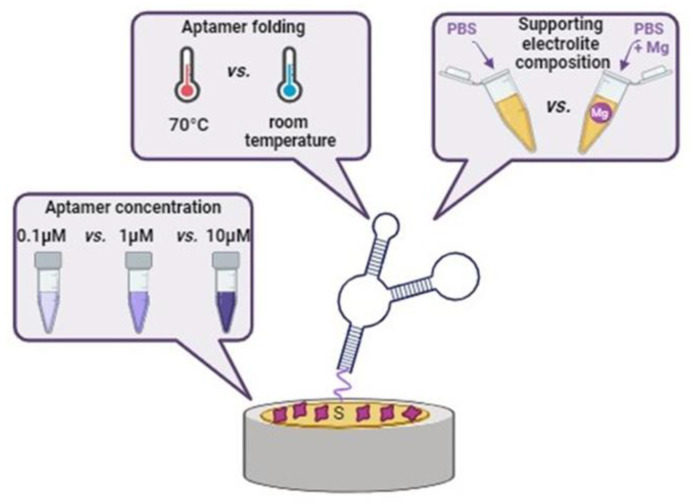
Illustration of variable parameters influencing the aptasensor’s sensitivity towards the RBP-4 protein.

Three different parameters influencing aptasensor sensitivity were studied: (i) the folding procedure of the aptamer, (ii) the presence or absence of Mg^2+^ in the modification and supporting electrolyte and (iii) the concentration of the aptamer in the modification solution (Figure 2).

In the first step, we tested the folding procedure and influence of Mg^2+^ ions present in the modification solution and in the supporting electrolyte, on the magnitude of the signal generated upon contact of modified electrodes with protein. Initially, the electrodes used in this experiment were modified in two steps: (i) with a mixture of 1 µM Apt-RBP-4 and 0.1 µM MCH during 3 h, and (ii) 1 mM of MCH during 30 min. The aptamer solution was either treated with the aptamer folding procedure or not. The prepared receptor layers were exposed to the solution containing 1 µg/mL RBP-4 protein. The results obtained (Table 1) clearly show that the folding procedure and Mg^2+^ ions influence the magnitude of the electrochemical signal. The highest relative changes in the Δ*E*, Δ*I* and Δ*R* parameters were observed for electrodes whose modifications and supporting electrolyte solutions contained MgCl_2_. An increase in the Δ*E* parameter in CV by about 35%, a decrease in the current expressed as Δ*I* by about −23%, and an increase in the Δ*R* of 68% were observed, respectively.

The binding of RBP-4 to specific aptamers influences the accessibility of [Fe(CN)_6_]^3−/4−^ to the surface of the electrode. As a consequence, the oxidation and reduction currents decreased, resulting in a higher difference in the potentials as compared with the value registered before the electrode came into contact with RBP-4. Further, blocking the electron transfer from the redox probe to the electrode influences the reduction in the peak current registered in SWV. Observations made during the CV and SWV experiments were further confirmed by the EIS results. The charge transfer resistance of the electrode, represented by the semicircle diameter in the Nyquist plot, significantly increased upon contact with the RBP-4 protein caused by the creation of a more insulating layer. All three techniques confirm the binding of RBP-4 protein to the surface of the electrodes to the greatest extent when electrodes were modified with aptamer treated with folding procedures and in the presence of Mg^2+^ ions in the modification and supporting electrolyte solutions.

### 3.2. Optimization of Aptamer Concentration in the Modification Solution

Within this part of the work, our aim was to control the surface density of aptamer molecules through the concentration of aptamer in the measurement solution in order to properly optimize the aptasensor sensitivity [38]. The sensitive detection requires a high surface density of the receptor, but on the other hand, it must be low enough to ensure proper folding and avoid steric hindrances [17]. Therefore, three different concentrations of the aptamer present in the modification solution were tested: either 0.1, 1.0 or 10.0 µM. As is presented in Table 2, decreasing the aptamer concentration in the modification solution (aptamer subjected to the folding procedure) up to 0.1 µM had the greatest impact on the analytical signals registered. It is clear that the Δ*E* parameter increased by 1.9-fold as compared to the 1 µM concentration of aptamer used. The same tendency was observed for the Δ*I* and Δ*R* parameters, where a 2.2-fold decrease and a 1.4-fold increase were observed, respectively. Therefore, as a further experiment, the following conditions were selected: 0.1 µM of aptamer concentration in the modification solutions based on the aptamer folding procedure, and a modification solution/supporting electrolyte containing PBS and MgCl_2_. 

The heterogeneous electron transfer rate constant (*k*_0_) and electron transfer coefficient (*α*) are the key parameters of the electrochemical performances of the electrodes modified in three different compositions: including 10.0, 1.0 and 0.1 µM of ssDNA aptamer and RBP-4 in solution [39,40]. The results are presented in Table S1 in the Supplementary Materials. The results clearly show that gold electrodes modified with 0.1 µM of aptamer in the solution showed the closest value to *α* = 0.5. 

In order to quantitatively determine ssDNA aptamer surface density at different concentrations in the modification solutions, 0.1, 1.0 and 10.0 µM, the redox cations [Ru(NH_3_)_6_]^3+^ were used for experiments [41]. These cations bind electrostatically to the anionic DNA phosphate backbone and allow for an electrochemical estimation of aptamer surface density. The density of thiol ssDNA aptamers was determined to be 3.1 ± 0.2 × 10^11^ mol/cm^2^ for a 0.1 µM concentration of RBP-4 aptamer in the immobilization solution, 4.8 ± 0.2 × 10^11^ mol/cm^2^ for 1.0 µM and 6.0 ± 0.5 × 10^11^ mol/cm^2^ for 10.0 µM (Table S1, Supplementary Materials) and shows a gradual increase in of surface density with the increasing concentration of the immobilization solution.

The kinetics of the modified gold electrodes’ reactions were studies based on SW voltammograms [42]. The values of the dimensionless electrode kinetic parameter (*K*) together with the standard rate constant of electron transfer (*k_s_*) are presented in Table S3 (Supplementary Materials).

### 3.3. Electrochemical Aptasensing of RBP-4 Protein

**Figure 3:**
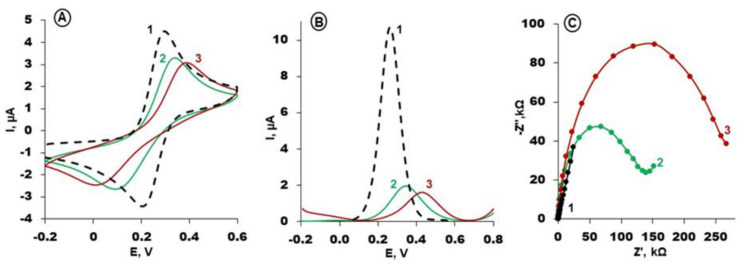
(**A**) Cyclic voltammograms (Scan rate: 0.1 V/s); (**B**) square wave voltammograms (Frequency: 50 Hz) and (**C**) Nyquist plots (bias potential of +0.17 V; frequency range from 0.1 Hz to 10 kHz) of: bare gold (1, black curves) and Apt/MCH modified gold electrode, before interaction with RBP-4 (2, green curves) and after interaction with 1 µg/mL of RBP-4 (3, red curves). Solution composition: 1 mM K_3_[Fe(CN)_6_]/K_4_[Fe(CN)_6_], 0.1 M PBS + 2 mM MgCl_2_ (pH 7.4).

The steps of gold electrode modification with an RBP-4 aptamer based on selected conditions were monitored with CV, SWV and EIS using [Fe(CN)_6_]^3−/4−^ as a redox probe in PBS with MgCl_2_. The obtained results are presented in Figure 3A–C. The oxidation/reduction current of a redox probe on a bare gold electrode is relatively high (Figure 3A,B, black curves) and the Nyquist plot appears as a straight line (Figure 3C, black curve). The modification of the gold electrode according to a two-step procedure using aptamer specific towards RBP-4 protein and MCH caused a decrease in the current registered in CV and SWV, assisted by the increase in resistance in the electrode in EIS (Figure 3, green curves). The formation of a self-assembled monolayer of thiol Apt-RBP-4 and MCH blocks the electron transfer of the redox probe to the surface of the gold electrode. Subsequently, the interaction of aptamer-modified electrodes with a specific protein, RBP-4, at a concentration of 1 µM caused a notable decrease in the Δ*E* and Δ*I* potential difference and current and an increase in resistance (Figure 3, red curves). 

In a further step, EIS was selected as an analytical technique for the observation of the interaction between the aptamer and RBP-4 through the electrochemical aptasensor. EIS is an effective technique frequently applied for controlling interfacial properties correlated with bio-recognition phenomena occurring at the interface between a solid electrode and a supporting electrolyte [43]. Additionally, taking into account the advantages of EIS, such as sensitivity, versatility and, most importantly, non-invasiveness, this technique was used to study interfacial features of ssDNA aptamer-modified gold electrode upon interaction with RBP-4. In general, more comprehensive data about a biosensing system can be obtained using EIS compared to information gained using more common voltammetric techniques [44,45].

**Figure 4:**
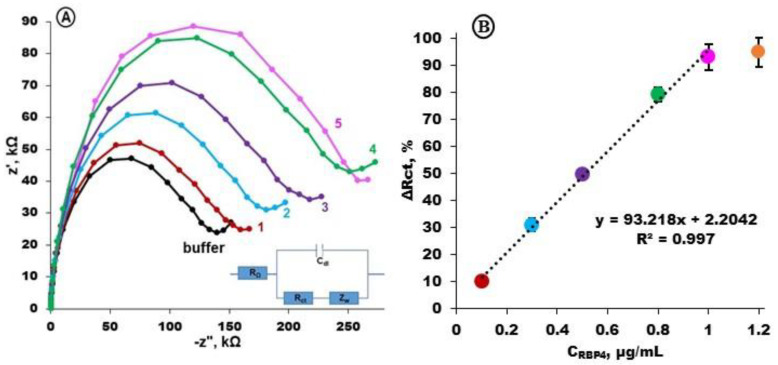
(**A**) Examples of electrochemical impedance spectra registered for gold electrodes modified with Apt-RBP-4/MCH (buffer, black) and next curves after 1h interaction with RBP-4 protein at particular concentrations: 0.1 μg/mL (1), 0.3 μg/mL (2), 0.5 μg/mL (3), 0.8 μg/mL (4) and 1.0 μg/mL (5). Solution composition: 1 mM K_3_[Fe(CN)_6_]/K_4_[Fe(CN)_6_] in 0.1 M PBS + 2 mM MgCl_2_, bias potential of +0.17 V; frequency range from 0.1 Hz to 10 kHz; (**B**) calibration curve represented as charge transfer resistance differences ΔR = (R_n_ –R_0_)/R_0_, [%] registered in the presence of 0.1; 0.3; 0.5; 0.8; 1.0; and 1.2 μg/mL RBP-4 protein in 0.1 M PBS + 2 mM MgCl_2_ (n = 3 ÷ 5).

To determine the calibration curve, electrochemical impedance spectra were registered before and upon the interaction of electrodes modified with Apt-RBP-4 with RBP-4 protein in different concentrations: 0.1, 0.3, 0.5, 0.8 and 1.0 µg/mL, respectively (Figure 4A). The subsequent increases in charge transfer resistance were observed, which were proportional to the RBP-4 protein concentration (Figure 4B). 

The kinetic parameters of the interaction between RBP-4 and ssDNA aptamer-immobilized on gold electrode were evaluated based electrochemical impedance spectroscopy data according the procedure in our previous paper [46]. The association constant *K_A_* of 2.0 ± 0.2 × 10^7^ M^−1^ was calculated by linearization of Langmuir isotherm according to the equation *K_A_C* = (*R_n_ − R*_0_)*/R*_0,_ where *C* is the concentration of redox probe in the solution, *R_0,_ R_n_* means the charge transfer resistance of layer without and with the presence of particular concertation of RBP-4 protein. The linear relationship of (*R_n_ − R*_0_)*/R*_0_ vs. *C* [*M*] is presented in Figure S1 in the Supplementary Materials. Therefore, the affinity interactions between the immobilized ssDNA aptamer and RBP-4 can be described by the dissociation constant *K_D_* of 5.0 ± 0.05 × 10^−8^ M. Then, the thermodynamic parameter of Δ*G* (change in Gibb’s free energy) was calculated using the Van’t Hoff equation [47] and was −38.16 ± 1.9 kJ/mol, which means the spontaneous interaction between ssDNA immobilized aptamer and RBP-4 protein (Table S2, Supplementary Materials).

The limit of detection (LOD) calculated based on the equation *LOD* = 3.3*σ/S* (where *σ* is the standard deviation of the signal response for the lowest concentration of RBP-4 and *S* is the slope of the calibration curve) [48] was 44 ng/mL. It was reported by Farjo and co-workers [49] that the serum level of RBP-4 for healthy people ranges from 10 to 50 µg/mL. However, the increase from 17 to 150 µg/mL of serum RBP-4 has been registered for patients with obesity, insulin resistance, type 2 diabetes or vascular disease [49]. Therefore, using the aptasensor presented in this work, RBP-4 protein can be easily detected in both physiological concentrations and in elevated concentrations associated with diseases.

A comparison of the electrochemical aptasensor for the detection of RBP-4 with other aptasensors has led to the following conclusions (Table 3). Most examples of aptasensors for RBP-4 detection concern spectroscopic methods of detection [9,10,11,12]. The LOD obtained in this work was better than SPR, ELAAS and colorimetric methods. But the detection time of 60 min. obtained in this work is competitive with other aptasensors whose detection time is up to 140 min. Only one example based on colorimetry, reported by Moabello and co-workers, estimated a 5-min detection time, but a much worse LOD [15]. It is clear that the electrochemical aptasensor presented in this work is beneficial in relation to the other aptasensors in terms of the LOD and detection time, as well as the cost and equipment requirements.

**Figure 5:**
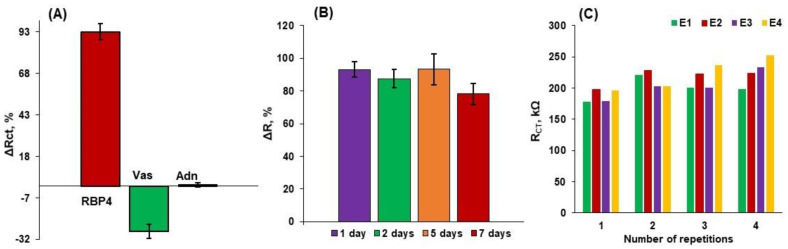
Results of (**A**) selectivity obtained by the relative changes of signals registered in EIS of the optimized system with the same concentration of RBP-4, vaspin and adiponectin of 1 µg/mL, (**B**) stability, and (**C**) repeatability and reproducibility studies obtained for the gold electrodes modified with Apt-RBP-4/MCH. Supporting electrolyte: 0.1 M PBS + 2 mM MgCl_2_ (n = 3 ÷ 5).

In order to estimate the practical use of aptasensor, selectivity, stability, repeatability and reproducibility (Figure 5) studies were conducted. The relative changes in signals registered in CV, SWV and EIS for electrodes modified with 0.1 µM of RBP-4 aptamer and subjected to the folding procedure upon interaction with 1 µg/mL of RBP-4, vaspin and adiponectin are shown in Table 4. 

Both parameters Δ*E* and Δ*I* are negligible for vaspin and adiponectin in comparison with RBP-4, whereas the Δ*R* parameter has been demonstrated to have a negative value for vaspin and no change for adiponectin compared to RBP-4 (Figure 5A). This further supports the idea that EIS is appropriate for studying the interaction between the aptamer deposited on the gold electrode and RBP-4 protein present in the analyzed sample. The obtained results allow us to conclude that the electrochemical aptasensor is capable of sensitive and selective detection of RBP-4. The stability studies show that this platform could maintain almost 100% Δ*R* during 5 days of storing, which is reduced by ~16% after 7 days of storing (Figure 5B). Subsequently, acceptable repeatability and reproducibility were estimated characterized by relative standard deviations of 5.7% and 4.7%, respectively (Figure 5C). 

The selectivity factor (*α_f_*) [50] was calculated based on the equation: 

*α_f_ =* (Δ*R*/*R*_0_)*_RBP_*_4_/(Δ*R*/*R*_0_)*_I_,* where (Δ*R*/*R*_0_)*_RBP_*_4_ and (Δ*R*/*R*_0_)*_Int_* are the normalized responses of aptamer to RBP-4 and particular interferents, respectively (Supplementary Materials). The values of *α_f_* calculated for vaspin and adiponectin were 3.4 and 91.9, respectively, highlighting that the aptasensor has high selectivity towards RBP-4 protein.

## 4. Conclusions

The main aim of this work was to investigate the effect of electrochemical aptasensor construction parameters on the analytical signal generated by the aptasensor towards RBP-4. Specifically, we examined the influence of magnesium ions present in the modification and measurement electrolyte, the folding procedure and aptamer concentration on aptasensor sensitivity. The important electrochemical parameters of the optimized sensing layers, including heterogeneous electron transfer rate constant (*k*_0_) and electron transfer coefficient (*α*), and density of the aptamer (*Γ_Apt_*), were also evaluated. The highest sensitivity was obtained for gold electrodes modified with 0.1 µM of Apt-RBP-4 in the presence of MgCl_2_ in both the modification and measurement solutions, subjected to folding procedure. 

Under the optimized conditions, the impedimetric aptasensor towards RBP-4 was characterized by a linear range between 100 and 1000 ng/mL and the LOD of 44 ng/mL. The obtained LOD allows for the detection of serum RBP-4 in healthy people and those suffering from diseases. The negligible response of aptasensor towards vaspin and adiponectin confirms aptasensor selectivity, which was further emphasized by selectivity factor (*α_f_*). The electrochemical aptasensor which is relatively simple in preparation, cost-effective and requiring simple equipment could be applied for detection and development of obesity, insulin resistance, type 2 diabetes or vascular disease. Further research related to the miniaturization of the system and testing in real samples is currently being conducted. 

## Figures and Tables

**Table 1:** The relative changes in signals registered in CV, SWV and EIS of a gold electrode modified in two steps: 1 µM Apt-RBP-4 and 0.1 µM MCH during 3 h and 1 mM MCH during 30 min, upon interaction with 1 µg/mL RBP-4 in the different composition of the modification solution and supporting electrolyte solutions and aptamer folding procedure.

Modification Solution/ Supporting Electrolyte	Aptamer Folding			Aptamer without Folding		
	Δ*E* [%]	Δ*I* [%]	Δ*R* [%]	Δ*E* [%]	Δ*I* [%]	Δ*R* [%]
PBS	9.7 ± 0.3	−9 ± 0.7	53.3 ± 5	14.6 ± 0.8	−7 ± 0.7	37.3 ± 5.5
PBS + MgCl_2_	**35.3 ± 2.9**	**−22.9 ± 0.7**	**67.5 ± 4.5**	39.7 ± 3.0	−19.6 ± 1.8	55.3 ± 4.3

The meaning of Δ*E* [%], Δ*I* [%] and Δ*R* [%] is explained in the experimental section. Optimal measurement conditions are shown in bold.

**Table 2:** The relative changes in signals registered in CV, SWV and EIS of a gold electrode modified in two steps depending on the concentration of thiolated aptamer Apt-RBP-4: either 0.1, 1.0 or 10.0 µM and 0.1 µM MCH during 3 h and 1 mM MCH during 30 min., upon interaction with 1 µg/mL RBP-4 in the different aptamer folding procedure. Supporting electrolyte: 0.1 M PBS + 2 mM MgCl_2_.

C_APT_ [µM]	Aptamer Folding			Aptamer without Folding		
	Δ*E* [%]	Δ*I* [%]	Δ*R* [%]	Δ*E* [%]	Δ*I* [%]	Δ*R* [%]
0.1	**67 ± 1.7**	**−51.7 ± 2.6**	**93.1 ± 4.8**	71 ± 4	−29.8 ± 1.7	46.2 ± 1.9
1	35.3 ± 2.9	−22.9 ± 0.7	67.5 ± 4.5	39.7 ± 3.0	−19.6 ± 1.8	55.3 ± 4.3
10	24.9 ± 1.2	−22.3 ± 1.1	58.4 ± 7.6	27.7 ± 1.6	−23.7 ± 1.8	35.8 ± 2.1

The meaning of Δ*E* [%], Δ*I* [%] and Δ*R* [%] is explained in the experimental section. Optimal measurement conditions are shown in bold.

**Table 3:** Comparison of the RBP-4 aptasensor parameters with other aptasensors used for RBP-4 detection.

Receptor	Method	LOD (ng/mL)	Linear Range (ng/mL)	Detection Time (min)	Ref.
ssDNA Aptamer	SPR	1580	200–500	140	[13]
	ELAAS	75	78–5000	120	[14]
	Chemiluminescence	95.1 × 10^−5^	0.001–2.0	120	[12]
	Colorimetric	1906	164 –5250	5	[15]
	EIS	44	100–1000	60	This work

SPR—Surface Plasmon Resonance; ELAAS—Enzyme Linked Aptamer Assays; EIS—Electrochemical Impedance Spectroscopy.

**Table 4:** The relative changes of signals registered in CV, SWV and EIS of gold electrode modified in two steps with 0.1 µM of Apt-RBP-4 and 0.1 µM MCH during 3 h and 1 mM MCH during 30 min. upon interaction with 1 µg/mL RBP-4, 1 µg/mL of vaspin and 1 µg/mL of adiponectin. Supporting electrolyte: 0.1 M PBS + 2 mM MgCl_2_ (n = 3 ÷ 5).

Analyte	ΔE [%]	ΔI [%]	ΔR [%]
RBP-4	67 ± 1.7	−51.7 ± 2.6	93.1 ± 4.8
Vaspin	7.8 ± 2.0	−13.8 ± 0.7	−27.2 ± 4.3
Adiponectin	10.7 ± 2.3	−1.5 ± 0.9	0.8 ± 1.4

The meaning of Δ*E* [%], Δ*I* [%] and Δ*R* [%] is explained in the experimental section.

## Data Availability

Data will be made available on request.

## Supplementary Materials

The following supporting information can be downloaded at: https://www.mdpi.com/article/10.3390/bios14020101/s1, Table S1: Electron transfer coefficients (*α*), electron transfer rate constants (*k*^0^) and surface coverage of the thiolated aptamer probe (*Γ_Apt_*) calculated for modified gold electrodes. Figure S1: The linear relationship of *(R_n_* − *R*_0_/*R_0_*) vs. *C* of RBP-4. Table S2: The association constant *K_A_*, dissociation constant *K_D_* and Gibbs free energy Δ*G* calculated for gold electrode modified with 0.1 µM of RBP-4 aptamer in solution. Table S3. The electrode kinetic parameter *K*, the standard rate constant of electron transfer *k_s_* calculated for gold electrode modified with 0.1 µM of RBP-4 aptamer in solution. Table S4. Summary of Δ*E*, Δ*I* and Δ*R_ct_* [%] values obtained using 1 mM [Ru(NH_3_)_6_]Cl_3_.
