# Supplementary material for: Electrochemical Aptasensing Platform for the Detection of Retinol Binding Protein-4
Source: Biosensors (Basel). 2024 Feb 16;14(2):101. doi: 10.3390/bios14020101 (PMC10887324; doi:10.3390/bios14020101)
Supplement: Supplementary file 1 [file biosensors-14-00101-s001.zip › biosensors-2824347-supplementary.pdf]

Supplementary Materials

Electrochemical Aptasensing Platform for the Detection of Retinol Binding Protein—4

Kamila Malecka-Baturo [1], Paulina Żółtowska [2], Agnieszka Jackowska [2], Katarzyna Kurządkowska-Adaszyńska [1] and Iwona Grabowska [1,*]

[1] Institute of Animal Reproduction and Food Research Polish Academy of Sciences, Tuwima 10, 10-748 Olsztyn, Poland; k.malecka@pan.olsztyn.pl (K.M.-B.); k.kurzatkowska-adaszynska@pan.olsztyn.pl (K.K.-A.)
[2] Department of Chemistry, University of Warmia and Mazury, Plac Łódzki 4, 10-721 Olsztyn, Poland; paulina.zoltowska2002@gmail.com (P.Ż.); agnieszka.jackowska123@gmail.com (A.J.)
* Correspondence: i.grabowska@pan.olsztyn.pl

1. The electroactive surface area of the gold working electrode A_{eas} was determined by oxygen adsorption measurement and was calculated using the Equations:

$$A_{eas} = \frac{Q_{Au}}{Q_H^S} = 0.43 \pm 0.03 \text{ cm}^2 \quad \text{(S1)}$$

$$Q_{Au} = \frac{A_{p,c}}{\upsilon} \quad \text{(S2)}$$

where $A_{p,c}$ is the charge of the gold oxide reduction of the gold working electrode, υ is the scan rate (0.1 V/s) and Q_H^S is the standards reference charge (0.00039 ± 0.00001 C/cm²) suggested for polycrystalline gold [1].

2. The roughness factor *RF* was calculated according to the Equation:

$$RF = \frac{A_{aes}}{A_{geom}} = 13.7 \pm 1.1 \quad \text{(S3) [2,3]}$$

where A_{geom}= 0.0314 cm²

3. The electron transfer coefficient α was calculated using the Equation:

$$\left|E_p - E_{p/2}\right| = \frac{1.857RT}{\alpha F} = \frac{47.7}{\alpha} \quad \text{(S4) [4,5]}$$

where E_p is peak potential, $E_{p/2}$ is the half wave potential, R is the universal gas constant (8.3145 J mol^{-1} K^{-1}), T is room temperature.

4. The heterogeneous electron transfer constant k^0 was calculated according to the Klingler–Kochi method:

$$k^0 = 2.18\left[\frac{\alpha DnF\nu}{RT}\right]^{1/2} e^{-\left[\frac{\alpha^2 nF}{RT}\right](E_{p,ox}-E_{p,red})} \quad \text{(S5) [4,5]}$$

where α was calculated from Eq. (S4), D is the diffusion coefficient of 5 mM $Fe(CN)_6^{3-/4-}$ (7.6× 10^{-6} cm² s^{-1}), F is Faraday constant (96500 C mol^{-1}), ν is scan rate (V s^{-1}), $E_{p,ox}$ and $E_{p,red}$ are oxidation and reduction peak potential, respectively.

5. The surface coverage of the thiolated aptamer probe Γ_{Apt} was calculated using the ability of electrostatic binding of redox cations, such as $[Ru(NH_3)_6]^{3+}$ to the anionic DNA phosphate backbone [6].

The surface concentration of $[Ru(NH_3)_6]^{3+}$ (Γ_{Ru}) was calculate according to the following Equation:

$$\Gamma_{Ru=} = \frac{Q}{nFA} \quad \text{(S6)}$$

where: n – number of electrons in redox reaction; F–Faraday constant [C/mol]; A–the area of the gold working electrode [cm²].

The aptamer surface densities Γ_{Apt} were calculated from the Equation:

$$\Gamma_{Apt} = \Gamma_{Ru}\frac{z}{m}N_A \quad \text{(S7)}$$

where: z–charge of the redox molecules; m–number of nucleotides; N_A–Avogadro's number.

Table S1. Electron transfer coefficients *(α)*, electron transfer rate constants *(k^0)* and surface coverage of the thiolated aptamer probe *(Γ_{Apt})* calculated for modified gold electrodes.

C_{Apt} [μM]	0.1	1	10
α	0.28 ± 0.01	0.20 ± 0.02	0.13 ± 0.01
k^0 [cm/s]	0.92 ± 0.01	0.99 ± 0.01	1.07 ± 0.01
Γ_{Apt} [mol/cm^2]	3.1 ± 0.2 × 10^{11}	4.8 ± 0.2 × 10^{11}	6.0 ± 0.5 × 10^{11}

6. The association constant (K_A) of the aptamer with RBP-4 was calculated by using a Langmuir isotherm approach [7].

$$k_A C = \frac{R_n - R_0}{R_0} \quad \text{(S8)}$$

where C is a concentration of molecules in the solution, and R_0 and R_n mean the charge transfer resistance of the layer without and in the presence of particular concentration of RBP-4, respectively.

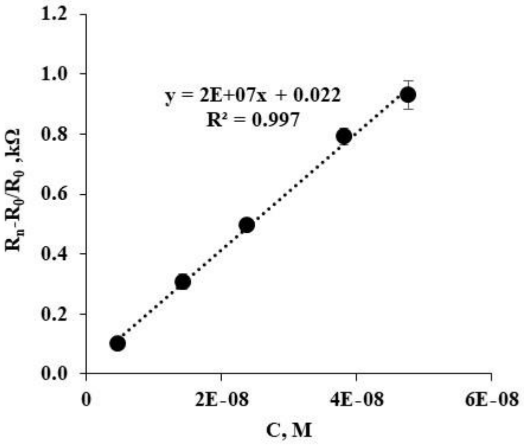

Figure S1: The linear relationship of *(R_n– R_0/R_0) vs. C* of RBP-4.

Based on the obtained results, the dissociation constant K_D was calculated according to the following relationship:

$$K_A = \frac{1}{K_D} \quad \text{(S9) [8]}$$

7. Gibbs free energy ΔG was calculated using Van't Hoff equation:

$$\Delta G = -RT\ln K_A \quad \text{(S10) [8]}$$

Table S2. The association constant K_A, dissociation constant K_D and Gibbs free energy ΔG calculated for gold electrode modified with 0.1 μM of RBP-4 aptamer in solution.

K_A [M^{-1}]	K_D [M]	ΔG [kJ/mol]
2.0 ± 0.02 ×10^7	5.0 ± 0.05 ×10^{-8}	−38.2 ± 1.9

8. The dimensionless electrode kinetic parameter *K* was calculated from the Equation:

$$\Delta E_{p/2} = -10.85\ln(K) + 80 \quad \text{(S11) [9]}$$

9. The standard rate constant of electron transfer k_s was calculated from the Equation:

$$K = k_s(fD)^{-0.5} \quad \text{(S12) [9]}$$

where *f* is frequency (50 Hz), *D* is the diffusion coefficient (7.6 $cm^2\ s^{-1}$).

Table S3. The electrode kinetic parameter *K*, and the standard rate constant of electron transfer k_s calculated for gold electrode modified with 0.1 μM of RBP-4 aptamer in solution.

**K**	**k_s [cm/s]**
0.255 ± 0.027	0.0054 ± 0.0005

10. The selectivity factor α_f of the proposed aptasensor was estimated using the Equation:

$$\alpha_f = \frac{\left(\frac{\Delta R_{ct}}{R_{ct,0}}\right)_{RBP4}}{\left(\frac{\Delta R_{ct}}{R_{ct,0}}\right)_{Int}} \quad \text{(S13) [10]}$$

where $\left(\frac{\Delta R_{ct}}{R_{ct,0}}\right)_{RBP4}$ and $\left(\frac{\Delta R_{ct}}{R_{ct,0}}\right)_{Int}$ are normalized responses of the Au/RBP-4 Apt/MCH to RBP-4 protein and interfering agents (Vaspin and Adiponectin).

<u>Experiments with positively charged redox-active indicator – hexaammineruthenium (II) chloride</u>

The electrochemical measurements using redox-active indicators with different physical and chemical characteristics are very helpful for evaluation of chemically modified electrodes. Therefore, aptasensor presented was also tested with positively charged redox-active indicator hexaammineruthenium (II) chloride and compared to the negatively charged of ferricyanide/ferrocyanide. Our results clearly showed that when the modified gold electrode was contacted with 1 μg/ml RBP-4, the ΔR_{ct} value decreased by 10% compared to an approximately 90% increase when ferri/ferro was used.

The obtained results are presented below:

Table S4. Summary of *ΔE, ΔI* and ΔR_{ct} [%] values obtained using 1 mM $[Ru(NH_3)_6]Cl_3$.

**C_{RBP4}, μg/mL**	**CV**	**OSWV**	**EIS**
	ΔE, %	ΔI, %	ΔR_{ct}, %
**0.1**	7.24 ± 0.15	-3.08 ± 1.48	4.59 ± 0.67
**0.5**	3.64 ± 0.09	-2.03 ± 1.35	-3.38 ± 0.99
**1**	3.56 ± 0.29	-1.20 ± 0.78	-9.77 ± 3.28

To explain this phenomenon, several parameters must be taken into account. Apart from ionic charge, these redox active indicators differ in size, molecular structure and standard electron-transfer rate constant (k^0) [11]. Just for example, $[Ru(NH_3)_6]Cl_3$ is characterized by ca. 10 times higher standard electron-transfer rate constant (k^0) than

$K_3[Fe(CN)_6]/K_4[Fe(CN)_6]$. Additionally, cationic redox markers such as $[Ru(NH_3)_6]Cl_3$ strongly interact with ssDNA oligonucleotides [12]. Moreover, the isoelectric point of RBP-4 appears to be in the pH range of 4.4 to 4.8 [13]. So, upon complex creation between aptamer and RBP-4, the surface becomes more negatively charged and further attraction occurs, not detected by electrochemical methods using $[Ru(NH_3)_6]Cl_3$. Additionally, $[Ru(NH_3)_6]^{3+}$ are only stable in oxidized state and electrochemical impedance spectroscopy biosensors using this redox probe are not frequently used [14]. Therefore, we recommend to use $K_3[Fe(CN)_6]$/ $K_4[Fe(CN)_6]$ for aptasensing of RBP-4.

References:

1. Zaki, M.H.M.; Mohd, Y.; Chin, L.Y. Surface properties of nanostructured gold coatings electrodeposited at different potentials. *J. Electrochem. Sci.* **2020**, *15*, 11401–11415.
2. Xie, X.; Holze, R. Electrode kinetic data: Geometric vs. real surface area. *Batteries* **2022**, *8*, 146.
3. García-Miranda Ferrari, A.; Foster, C.W.; Kelly, P.J.; Brownson, D.A.C.; Banks, C.E. Determination of the electrochemical area of screen-printed electrochemical sensing platforms. *Biosensors* **2018**, *8*, 53.
4. Trachioti, M.G.; Lazanas, A.C.; Prodromidis, M.I. Shedding light on the calculation of electrode electroactive area and heterogeneous electron transfer rate constants at graphite screen-printed electrodes. *Microchim Acta* **2023**, *190*, 251.
5. Forouzanfar, S.; Alam, F.; Pala, N.; Wang, Ch. Highly sensitive label-free electrochemical aptasensors based on photoresist derived carbon for cancer biomarker detection. *Biosens. Bioelectron.* **2020**, *170*, 112598.
6. Ge, B.; Huang, Y.-Ch; Sen, D.; Yu, H.-Z. Electrochemical investigation of DNA-modified surfaces: From quantitation methods to experimental conditions. *J. Electroanal. Chem.* **2007**, *602*, 156–162.
7. Szymańska, I.; Radecka, H.; Radecki, J.; Kaliszan, R. Electrochemical impedance spectroscopy for study of amyloid β-peptide interactions with (−)nicotine ditartrate and (−)cotinine. *Biosens. Bioelectron.* **2007**, *22*, 1955-1960.
8. Sikarwar, B.; Singh, V.V.; Sharma, P.K.; Kumar, A.; Thavaselvam, D.; Boopathi, M.; Singh, B.; Jaiswal, Y.K. DNA-probe-target interaction based detection of Brucella melitensis by using surface plasmon resonance. *Biosens. Bioelectron.* **2017**, *87*, 964–969.
9. Khalifa, M.M.; Elkhawaga, A.A.; Hassan, M.A.; Zahran, A.M.; Fathalla, A.M.; El-Said, W.A.; El-Badawy O. Highly specific Electrochemical Sensing of Pseudomonas aeruginosa in patients suffering from corneal ulcers: A comparative study. *Sci Rep* **2019**, *9*, 18320.
10. Tabrizi, M.A.; Acedo, P. Highly sensitive aptasensor for the detection of SARS-CoV-2-RBD using aptamer-gated methylene blue@mesoporous silica film/laser engraved graphene electrode. *Biosens. Bioelectron.* **2022**, *215*, 114556.
11. Chailapakul, O.; Crooks, R.M. Interactions between organized, surface-confined monolayers and liquid-phase probe molecules. 4. synthesis and characterization of nanoporous molecular assemblies: mechanism of probe penetration. *Langmuir* **1995**, *11*, 1329–1340.
12. Steichen, M.; Doneux, T.; Buess-Herman, C. On the adsorption of hexaammineruthenium (III) at anionic self-assembled monolayers. *Electrochim. Acta* **2008**, *53(21)*, 6202–6208.
13. Peterson, P.A.; Berggård, I. Isolation and Properties of a Human Retinol-transporting Protein, *The Journal of Biological Chemistry* **1971**, *246*, 25–33.
14. Schrattenecker, J.D.; Heer, R.; Melnik, E.; Maier, T.; Fafilek, G.; Hainberger, R. Hexaammineruthenium (II)/(III) as alternative redox-probe to Hexacyanoferrat (II)/(III) for stable impedimetric biosensing with gold electrodes. *Biosens. Bioelectron.* **2019**, *127*, 25–30.
